# Programmable Low-Power Low-Noise Capacitance to Voltage Converter for MEMS Accelerometers

**DOI:** 10.3390/s17010067

**Published:** 2016-12-30

**Authors:** Guillermo Royo, Carlos Sánchez-Azqueta, Cecilia Gimeno, Concepción Aldea, Santiago Celma

**Affiliations:** 1 Group of Electronic Design—Aragón Institute of Engineering Research, Universidad de Zaragoza, 50009 Zaragoza, Spain; csanaz@unizar.es (C.S.-A.); caldea@unizar.es (C.A.); scelma@unizar.es (S.C.); 2 Institute of Information and Communication Technologies, Electronics and Applied Mathemathics, Université Catholique de Louvain, 1348 Louvain-la-Neuve, Belgium; cecilia.gimenogasca@uclouvain.be

**Keywords:** accelerometer, capacitive sensing, microelectromechanical systems (MEMS), synchronous demodulation, transimpedance amplifier

## Abstract

In this work, we present a capacitance-to-voltage converter (CVC) for capacitive accelerometers based on microelectromechanical systems (MEMS). Based on a fully-differential transimpedance amplifier (TIA), it features a 34-dB transimpedance gain control and over one decade programmable bandwidth, from 75 kHz to 1.2 MHz. The TIA is aimed for low-cost low-power capacitive sensor applications. It has been designed in a standard 0.18-μm CMOS technology and its power consumption is only 54 μW. At the maximum transimpedance configuration, the TIA shows an equivalent input noise of 42 fA/$\sqrt{\text{Hz}}$ at 50 kHz, which corresponds to 100 μg/$\sqrt{\text{Hz}}$.

## 1. Introduction

Capacitive sensors based on microelectromechanical systems (MEMS) are one of the most used accelerometers, since they offer high sensitivity, low power consumption and an excellent noise performance. They are also inexpensive and can be used in a wide range of applications, from the automotive industry, e.g., on assisted stabilization systems, to videogames and consumer electronics, such as smartphones or tablets [1,2,3].

The basic operational principle of capacitive accelerometers consists of a proof mass and fixed electrodes with a small gap between them, effectively creating a capacitor, with a capacitance, *C*_0_, ranging from 100 fF, up to a few pF. The acceleration causes the displacement of the proof mass, changing the gap distance. Therefore, a variation on the capacitance between the proof mass and the fixed electrodes is generated, and the acceleration is then measured from the capacitance variations. However, measuring these capacitances requires a high-sensitivity, highly-linear sensor interface, as their variations, *ΔC*, tend to be extremely small, usually less than 1 fF. Moreover, the parasitic effects are of great importance, since they can be as large as the sensor capacitance, which greatly affects the sensitivity of the measurement. MEMS accelerometers feature very low noise, with values at about 20 μg/$\sqrt{\text{Hz}}$ for bulk micromachined accelerometers and about 300 μg/$\sqrt{\text{Hz}}$ for surface micromachined capacitive structures, which can be fabricated at a significantly lower cost [4,5,6,7]. Nevertheless, in either case, in order to take advantage of the extremely low thermal noise of the MEMS accelerometers, an ultra-low noise sensor interface is needed.

Several techniques can help reduce the impact of the parasitic capacitances. For example, the bootstrapping one, where the current flow through the parasitic capacitors is virtually eliminated by ensuring that there is no voltage difference over them [1]. However, a highly accurate unity gain amplifier and a guard electrode surrounding the measurement electrode are needed. In many cases, manufacturing such guard electrodes is very difficult or even impossible. A good alternative to bootstrapping is the use of a transimpedance amplifier with a very low input impedance. The transimpedance amplifier (TIA) measures the current through the capacitor and practically eliminates the voltage variations at the input node, thus minimizing the effect of the parasitic capacitances without needing any guard electrode. Open loop TIAs based on the common gate or the regulated cascode input stage can show a very low input impedance, which makes them suitable for high speed applications [8,9,10]. However, closed loop TIAs can achieve a much higher transimpedance and perform a better performance in terms of input noise, being more suitable for low-noise sensor applications [11,12]. 

This work focuses on the design of the capacitance to voltage converter to improve its noise performance and to provide frequency response adaptability. The capacitance-to-voltage converter (CVC) is based on a transimpedance amplifier which features programmable gain and bandwidth, so that it can be adapted to different capacitive accelerometers.

The paper is organized as follows: Section 2 provides a general description of the proposed CVC and the blocks that it consists of, as well as the techniques that have been employed to perform the acceleration measurement. It also introduces the topology selected for the design of the TIA and the implementation of a programmability of the gain and bandwidth. Finally, Section 3 presents the results of this work and Section 4 provides the main conclusions.

## 2. Capacitance to Voltage Converter

**Figure 1:**
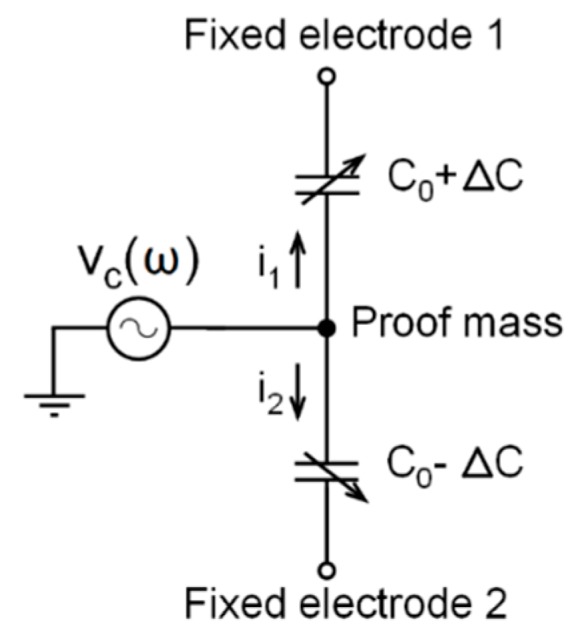
Electrical model of a capacitive accelerometer based on a microelectromechanical system (MEMS).

Figure 1 shows a simplified electrical model of a MEMS capacitive sensor. Measuring the current flow through the sensor is needed to measure the capacitance between the proof mass and the fixed electrodes. The current flow through a capacitor, *i*, is given by:

(1) $$i=C\frac{\partial V}{\partial t}+V\frac{\partial C}{\partial t}$$

where *C* is the capacitance and *V* the voltage over the capacitor. The first term is the displacement measurement and the second one is known as the rate-of-change measurement.

Typically, only one of these terms is measured, as it is usually much larger than the other one, depending on the application. The rate-of-change measurement is rarely used for measuring acceleration, as it is only measurable at higher frequencies. However, it can be used for other applications, such as resonators, that operate at higher frequencies. In [13] a capacitive readout interface for gravimetric chemical gas sensors based on a MEMS resonator is presented. This measurement mode can also be used to design oscillators, as in [14], where a 100-MHz oscillator based on a MEMS resonator is presented.

**Figure 2:**
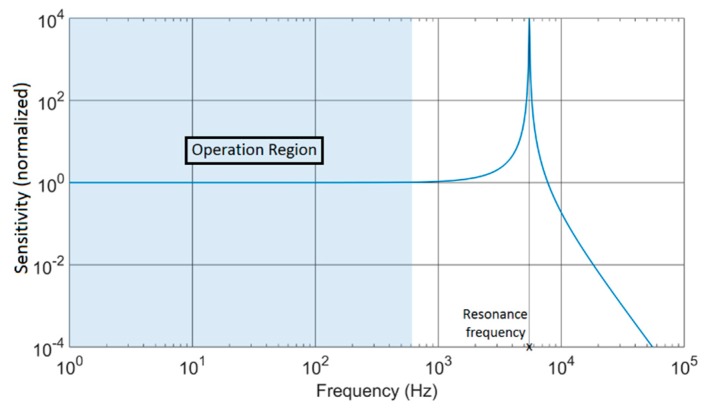
Mechanical frequency response of a capacitive MEMS accelerometer.

For acceleration measurement applications, the displacement measurement is employed, as the frequency range of the acceleration usually covers the range from 0 to a few hundreds of Hz, which coincides with the typical linear range of the mechanical response of a MEMS (Figure 2). This response depends on the physical parameters of the MEMS: proof mass, spring constant, damping factor, size, etc. [5,15,16,17]. Different MEMS will have a completely different mechanical response in terms of sensitivity, linear region or resonant frequency. Therefore, a sensor interface with a programmable response, adaptive to the mechanical MEMS response enhances the quality of the measurement, optimizing noise performance and sensitivity.

Measuring the displacement of the proof mass, that is, the first term in Equation (1), requires a high frequency measurement signal, $v_{c}=a\cdot \text{sin}\left(\omega_{c}t\right)$, for several reasons. Firstly, for higher frequencies, the current flow through the capacitive sensor will be higher, thus increasing the overall sensitivity. Secondly, this frequency cannot be close to the mechanical resonance frequency of the MEMS, since it could begin to oscillate. Finally, if this signal frequency is much higher than the frequency of the capacitive variations, the second term in Equation (1) can be neglected.

**Figure 3:**
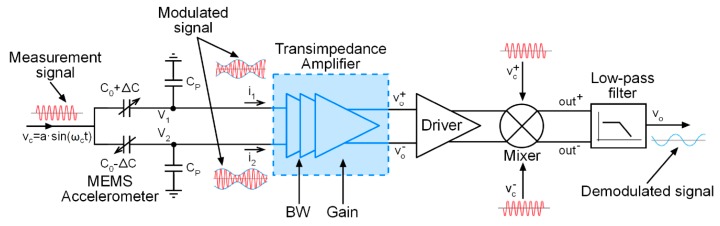
Conceptual scheme of the open loop synchronous demodulator.

Figure 3 shows the overall scheme of the open loop synchronous demodulator. The measurement signal, $v_{c}$, acts as a carrier signal, while the capacitance of the sensor modulates the amplitude of the current flowing through the capacitor. However, as the capacitance variations tend to be extremely small, of the order of fF, the current flowing through the sensor is as well very small, of the order of few pA. Therefore, to obtain a measurable signal, a high-gain low-noise transimpedance amplifier is required. The TIA must feature low noise and low input impedance. With a low input impedance the TIA keeps the input nodes $V_{1}$ and $V_{2}$ at the ground potential, improving the effect of the bootstrapping technique, as the TIA practically eliminates the effect of the parasitic capacitances by suppressing the voltage variations at the input nodes. In this work, the CVC has been proposed to be used in an open loop synchronous demodulator for capacitive sensor interfaces, however, it could be used in closed loop counterparts based on the force-feedback technique. In this way, more precision can be achieved if a suitable controller is included to steer an actuation mechanism that forces the proof mass back to its rest position.

To demodulate the high frequency measurement signal and transfer it back to the baseband, a double-balanced Gilbert cell has been employed. The Gilbert cell shows high conversion gain, high linearity, and achieves good port-to-port isolation with wide bandwidth and low power consumption. In the last stage, the high frequency components are filtered, employing a low-pass filter that isolates the baseband, to properly measure the capacitance variations.

### Transimpedance Amplifier Design

**Figure 4:**
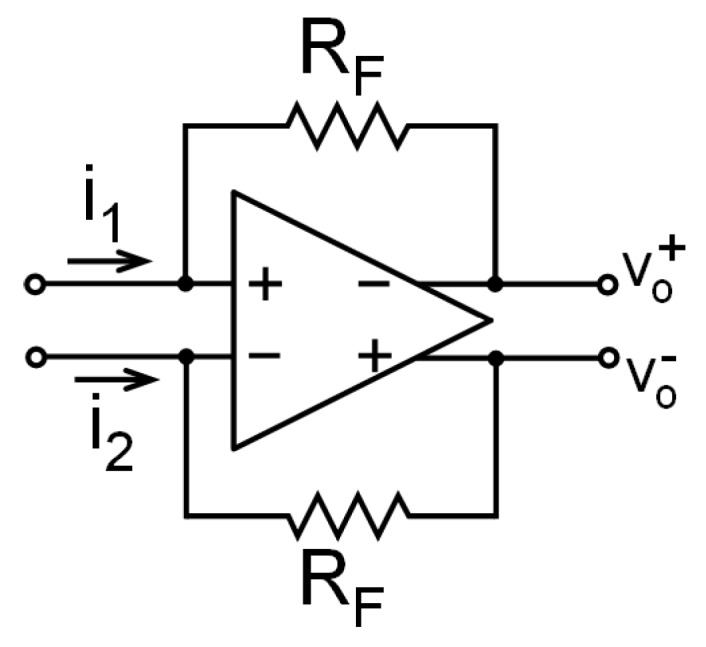
Differential shunt-shunt feedback transimpedance amplifier.

In this work a differential shunt-shunt feedback topology, shown in Figure 4, has been chosen to implement the TIA. It consists of a high-gain voltage amplifier with a negative feedback loop and it can achieve a high transimpedance, low input impedance, and low equivalent input noise (EIN). Using Equation (1) we obtain that the TIA amplifies the current and provides a measurable voltage signal, proportional to the capacitance variation:

(2) $$i_{1,2}≃\left(C_{0}\pm \Delta C\right)\frac{\partial v_{C}}{\partial t}$$

(3) $$\begin{array}{l} v_{o}^{+}-v_{o}^{-}=R_{T}\cdot \left(i_{1}-i_{2}\right)\\ v_{o}^{+}-v_{o}^{-}=2\Delta C\cdot R_{T}\frac{\partial v_{c}}{\partial t} \end{array}$$

where $R_{T}$ is the transimpedance of the TIA.

The amplitude of the signal $v_{o}^{+}-v_{o}^{-}$ carries the information of the measured acceleration. In order to maximize the sensitivity, the TIA must show high transimpedance, but also a bandwidth wide enough to contain the frequency of the carrier, so that the signal will not suffer attenuation. Nevertheless, as the bandwidth increases, more noise is integrated, thus reducing the signal-to-noise ratio (SNR). This tradeoff indicates that there is an optimum bandwidth, for which the sensitivity and SNR are optimized. This bandwidth is conditioned by the measurement signal frequency. Moreover, since this frequency is chosen depending on the MEMS resonance frequency, the optimum bandwidth will be a function of the mechanical MEMS response. In this work, we propose a TIA with programmable gain and bandwidth to optimize the sensitivity and the SNR for a wide range of mechanical MEMS responses.

As has been mentioned above, the parasitic capacitive effects must be compensated to maximize the sensitivity of the measurement. For this reason a transimpedance amplifier that suppresses the voltage variations at the input nodes is used, so that, ideally, there is no current flow through the parasitic capacitors. Therefore, all of the current through the sensor can be measured. The shunt-shunt feedback topology is the most used TIA configuration, as it achieves better linearity than common-gate based configurations, which also tend to be noisier. The proposed shunt-shunt feedback TIA consists of a fully differential voltage amplifier and a negative resistive loop. In a second-order approximation, where the differential voltage amplifier presents a DC gain, $A_{d}$, and a dominant pole frequency, $\omega_{A}$, the transfer function of the feedback TIA is given by:

(4) $$R_{T}=-\frac{2R_{F}}{1+\frac{2R_{F}C_{in}}{A_{d}}s+\frac{2R_{F}C_{in}}{A_{d}\omega_{A}}s^{2}}$$

where $C_{in}$ is the total input capacitance. To better describe the behavior of this second-order response, we study the quality factor, *Q*, which from Equation (4), can be calculated as:

(5) $$Q=\sqrt{\frac{A_{d}}{2R_{F}C_{in}\omega_{A}}}$$

**Figure 5:**
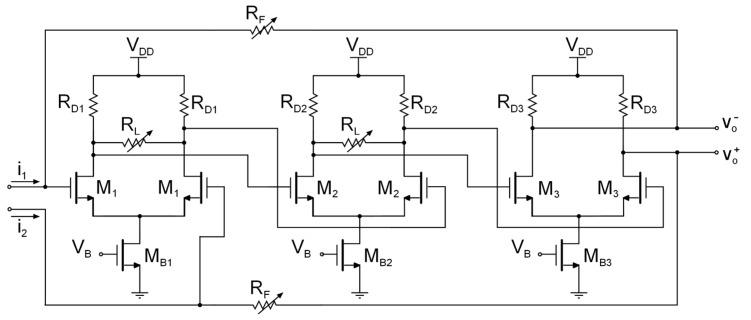
Simplified schematic circuit of the proposed transimpedance amplifier.

**Figure 6:**
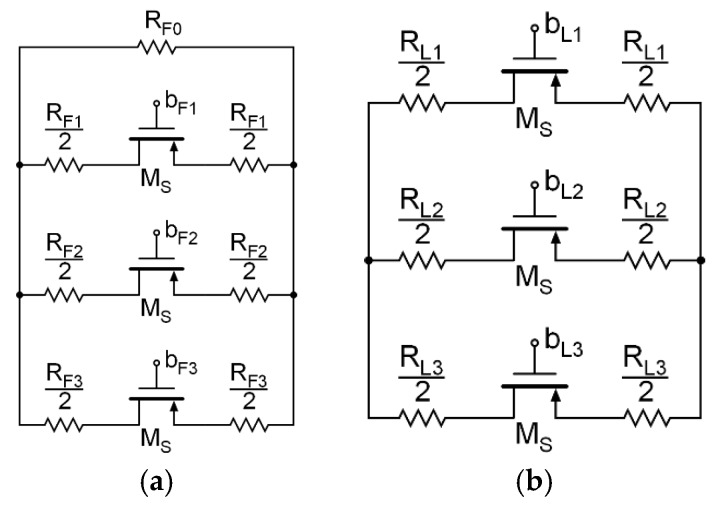
Implementation of (**a**) the feedback resistors, $R_{F}$, and (**b**) the load resistors, $R_{L}$.

According to Equation (4), the DC transimpedance is determined by $R_{F}$. With variable feedback resistors, the transimpedance can be programmed to optimize the sensitivity and SNR of the CVC. Nevertheless, the quality factor must also be considered when implementing a transimpedance gain control, as *Q* depends on the feedback resistance, and its value is restricted to certain values to maintain stability. It is well known that if *Q* > $1/\sqrt{2}$ there will be peaking in the frequency response, thus increasing the distortion of the signal and, more critically, the system could start to oscillate. Therefore, in order to maintain *Q* < $1/\sqrt{2}$, the open loop differential voltage gain, $A_{d}$, and the feedback resistor, $R_{F}$, have to be simultaneously controlled [18,19]. The proposed TIA, shown in Figure 5, performs the simultaneous control of both the feedback resistance and the open loop voltage gain. The transimpedance control has been implemented with a variable feedback resistor which consists of an array of four resistors connected in parallel, and programmable with a three-bit thermometer code, b_F,i_ (Figure 6a).

In order to implement the variable open loop gain, a voltage amplifier that allows an open loop gain control has been designed. It consists of three cascaded differential pairs with variable load resistors added to the first two pairs. The voltage gain, $A_{p}$ of a differential pair can be written as:

(6) $$A_{p}=g_{m}\left(R_{D}∥\frac{R_{L}}{2}\right)$$

where $g_{m}$ is the pair transonductance, and $R_{D}$ and $R_{L}$ the drain and load resistors, respectively. PMOS (p-type metal-oxide-semiconductor) transistors are commonly used to implement the drain resistors, as they require smaller area and can be externally controlled. However, active PMOS loads need a common mode feedback (CMFB) loop to stabilize the voltage at the output of the differential pair, thus demanding a higher power consumption, and triode PMOS loads exhibit a poor linear behavior, which highly degrades the overall linearity. In this work, inspired by high-frequency amplifiers, where CMFB loops are hard to implement due to stability issues, high-resistivity polysilicon resistors have been employed to achieve better linearity and lower power consumption.

In a similar way to the transimpedance gain control, the load resistors have been implemented using an array of three resistors in parallel, programmable with a three-bit thermometer code, b_L,i_ (Figure 6b), providing full programmability to the TIA, without modifying the common mode output voltage, as there is no DC current flow through these resistors. The component values and the aspect ratio of the transistors are shown in Table 1.

## 3. Discussion

The transimpedance amplifier has been designed in a standard 0.18-μm CMOS technology with a single 1.8-V voltage supply. For this work, we have used the model of a surface-micromachined comb-finger structure of 2-μm gap and resonance frequency of 5.5 kHz, with a nominal capacitance $C_{0}$ of 1 pF with parasitic capacitances $C_{P}$ of 2 pF. With this model of the MEMS accelerometer, the sensitivity provided by the sensor can be obtained from the following expression:

(7) $$S_{T}=\frac{\Delta C}{\Delta \ddot{x}}=\frac{\Delta x}{\Delta \ddot{x}}\cdot \frac{\Delta C}{\Delta x}$$

with *x* being the displacement of the proof mass. Considering a linear behavior, and at low frequency operation, it can be approximated by [20]:

(8) $$S_{T}=\frac{1}{\omega_{r}^{2}}\cdot \frac{C_{0}}{x_{0}}$$

with $x_{0}$ being the gap distance and $\omega_{r}$ the resonance frequency. From Equation (8) we obtain a sensitivity of 4.2 fF/g. The measurement frequency, $\omega_{c}$, is chosen to be of 50 kHz, so that it is roughly one order of magnitude greater than the resonance frequency.

**Figure 7:**
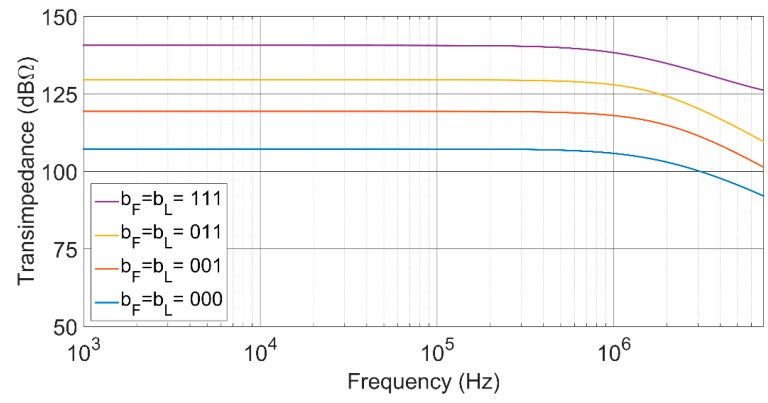
Frequency response showing the transimpedance gain programmability with a double control of the feedback resistor and the open loop gain. The bandwidth is almost constant at 1.2 MHz.

**Figure 8:**
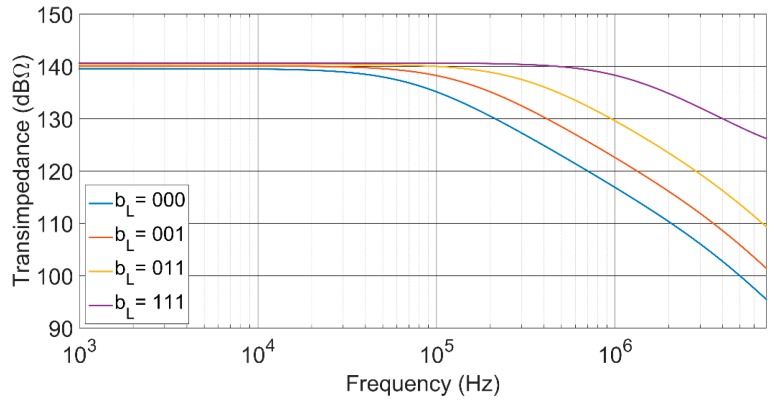
Frequency response with a single control of the open loop gain. The transimpedance gain is almost constant, at 140 dBΩ with feedback control word, b_F_ = 111.

As shown in Figure 7, the proposed TIA performs a 34-dBΩ transimpedance control range, while maintaining an almost constant bandwidth of around 1.2 MHz. To achieve this gain range keeping a flat frequency response, a simultaneous control of the feedback and load resistors has been made. Moreover, Figure 8 shows that with a single control of the open loop gain of the voltage amplifier, the transimpedance can be fixed at its maximum value, but the bandwidth can be tuned over more than a decade, from 75 kHz to 1.2 MHz.

**Figure 9:**
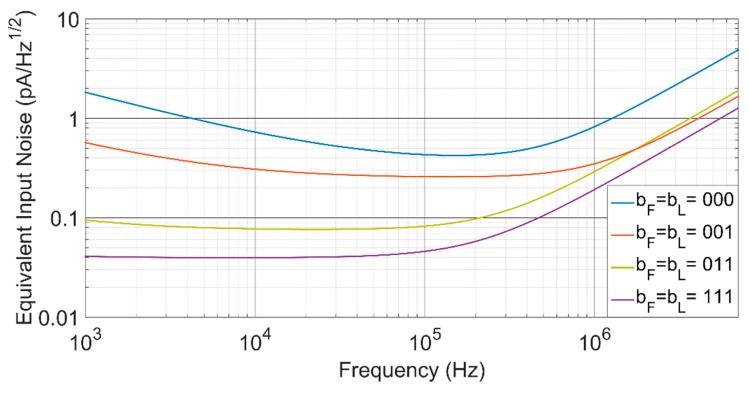
Equivalent input noise figure for the four gain configurations with 1.2 MHz bandwidth.

With a power consumption of only 54 μW, the TIA achieves a maximum sensitivity of 1 mV/fF, which corresponds to a capacitive sensitivity of 4.2 mV/g and presents a low equivalent input noise of only 42 fA/$\sqrt{\text{Hz}}$, at the maximum transimpedance configuration of 10 MΩ and 73-dB open loop gain (Figure 9, b_F_ = b_L_ = 111), or equivalently 100 μg/$\sqrt{\text{Hz}}$ at 50 kHz. Table 2 summarizes the main results and compares with recent published capacitance to voltage converters [17,21,22,23]. These CVCs are based on current measuring with a sensor interface based on a TIA, excepting [17], where a folded cascade operational amplifier is used configured as voltage amplifier, along with a diode-connected sub-threshold NMOS transistor to bias the input nodes. It also requires post-amplifying stages to increase sensitivity, including a variable gain amplifier to achieve a 12-dB gain control, which makes it a power-hungry circuit. A capacitive TIA is presented in [21], showing a 56-MΩ transimpedance and a 1.8-MHz bandwidth, which features no gain control and exhibits a higher noise level than that reported in this work. Structures reported in [22,23] are both based on resistive TIAs, providing transimpedance gains up to 25 MΩ in [22] and 22 MΩ in [23]. Both perform a transimpedance gain control and implement the feedback resistor using a T-network pattern, which reduces the overall size of the resistor, but can significantly increase noise. Moreover, the bandwidth in [22] decreases with the transimpedance, while an orthogonal gain and bandwidth control is achieved in the TIA proposed in this work. This full programmability of gain and bandwidth provides a much better adaptability of the frequency response to the mechanical frequency response of different MEMS.

**Figure 10:**
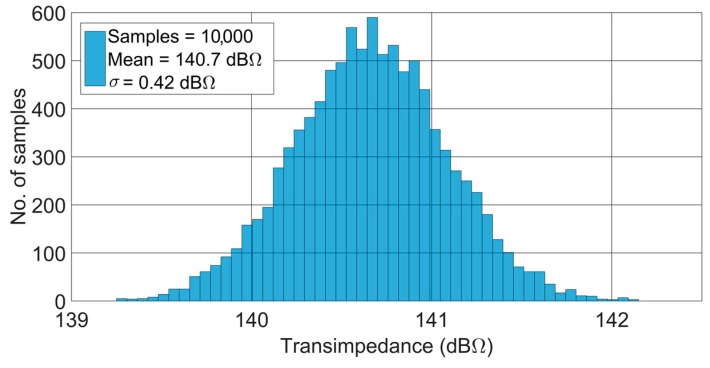
Monte Carlo simulation results for transimpedance gain for the maximum transimpedance configuration, 140 dBΩ, showing a standard deviation, σ, of 0.42 dBΩ.

**Figure 11:**
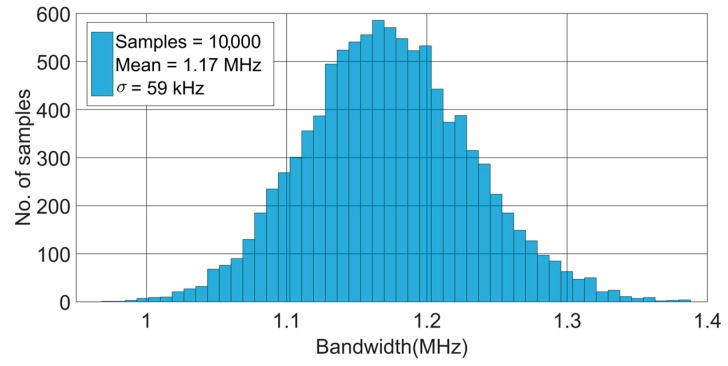
Monte Carlo simulation results for bandwidth for the maximum transimpedance configuration, 140 dBΩ.

Furthermore, Monte Carlo simulations have also been carried out for process variations and mismatch. The Monte Carlo analysis shows a statistical distribution of the chip performance before fabrication, which is of great importance, since the technological parameters can experience strong variations that may considerably deteriorate the overall performance. Figure 10 and Figure 11 present the Monte Carlo histograms for the transimpedance and bandwidth of the TIA, both of them obtained for the maximum gain configuration. The results obtained confirm the robustness of the design against process variations, with standard deviations of about 5% of the nominal value.

## 4. Conclusions

In this work, a capacitance-to-voltage converter for MEMS accelerometers has been presented. It is based on a new concept of a fully-differential transimpedance amplifier and achieves a 34-dB programmable gain range and over one decade programmable bandwidth. It has been designed in a standard 0.18-μm CMOS technology, and it is aimed for a differential surface-micromachined comb-finger capacitive accelerometer. The TIA achieves an equivalent input noise of 42 fA/$\sqrt{\text{Hz}}$ (100 μg/$\sqrt{\text{Hz}}$) at 50 kHz at the maximum transimpedance configuration. Its programmability allows its use in a wide range of capacitive MEMS devices, adapting the frequency response to the mechanical MEMS response. The presented TIA is compatible with low-power applications, with a power consumption of only 54 μW, and is robust against process variations, with a 5% standard deviation in gain and bandwidth.

## Figures and Tables

**Table 1:** Component values and transistor aspect ratios for the proposed transimpedance amplifier.

$M_{1}\left(\frac{W}{L}\right)$	$M_{2}\left(\frac{W}{L}\right)$	$M_{3}\left(\frac{W}{L}\right)$	$M_{B1-3}\left(\frac{W}{L}\right)$	$M_{S}\left(\frac{W}{L}\right)$	$R_{D1-3}$	$V_{B}$
20 μm/1 μm	20 μm/0.5 μm	10 μm/1 μm	10.5 μm/4 μm	20 μm/0.18 μm	180 kΩ	600 mV
$R_{F0}$	$R_{F1}$	$R_{F2}$	$R_{F3}$	$R_{L1}$	$R_{L2}$	$R_{L3}$
5 MΩ	1.1 MΩ	380 kΩ	100 kΩ	33 kΩ	48 kΩ	45 kΩ

**Table 2:** Summary of the performance and comparison with recently published works.

	[17] ^1^	[21] ^2^	[22] ^1^	[23] ^2^	This Work ^2^
Technology	0.35 μm CMOS	0.18 μm CMOS	0.6 μm CMOS	0.35 μm CMOS	0.18 μm CMOS
Supply Voltage	3.3 V	1.8 V	3 V	5 V	1.8 V
Minimum Input Noise	54 μg/$\sqrt{\text{Hz}}$	65 fA/$\sqrt{\text{Hz}}$	88 fA/$\sqrt{\text{Hz}}$	63 fA/$\sqrt{\text{Hz}}$ *	42 fA/$\sqrt{\text{Hz}}$
Capacitive Sensitivity	1450 mV/fF* (18 mV/fF without PA)	-	25 mV/fF *	3.3 mV/fF	1 mV/fF
Power Consumption	5.1 mW	436 μW	400 μW	-	54 μW
Type of Sensing Interface	Voltage Amplifier	Capacitive TIA	Resistive TIA	Resistive TIA	Resistive TIA
Gain	−9 to +2 dB	56 MΩ	1.6–25 MΩ	2–22 MΩ	0.15–10 MΩ
Bandwidth	8.6 MHz	1.8 MHz	200 kHz *	200 kHz *	75 kHz–1.2 MHz
Application	Accelerometer	Resonator/Oscillator	Gyroscope	Gyroscope	Accelerometer

^1^ Experimental results; ^2^ Simulation results; * Calculated from paper.
